# Secretion of Legumain Increases in Conditioned Medium from DJ-1-Knockout Cells and in Serum from DJ-1-Knockout Mice

**DOI:** 10.2174/1874091X01812010029

**Published:** 2018-02-28

**Authors:** Takuya Yamane, Izumi Kato-Ose, Tatsuji Sakamoto, Yoshihisa Nakano

**Affiliations:** 1 Center for Research and Development Bioresources, Research Organization for University-Community Collaborations, Osaka Prefecture University, Sakai, Osaka 599-8570, Japan; 2 Department of Applied Life Sciences, Graduate School of Life and Environmental Sciences, Osaka Prefecture University, Sakai, Osaka 599-8531, Japan; 3 Faculty of Pharmaceutical Sciences, Hokkaido University, Kita-ku, Sapporo 060-0812, Japan

**Keywords:** Legumain, DJ-1, Secretion, p53, Western blotting, Knockout mice, Gene

## Abstract

**Background:**

Asparaginyl endopeptidase, also known as legumain (EC 3.4.22.34) shows strong activity in the mouse kidney. Legumain is also highly expressed in tumors. DJ-1/PARK7 is a Parkinson’s disease- and cancer-associated protein. DJ-1 is a coactivator of various transcription factors. Recently, we reported that transcription of the legumain gene is regulated by p53 through DJ-1.

**Methods:**

We measured the secretion levels of legumain in a conditioned medium of DJ-1 knockout cells and in serum from DJ-1 knockout mice using Western blotting and ELISA. We performed immunocytochemical staining of legumain to examine the localization of legumain in DJ-1-knockout cells.

**Results:**

We found that the secretion levels of legumain were increased in the conditioned medium of DJ-1-knockout cells and in serum from DJ-1-knockout mice. Dot structures of legumain were also increased in DJ-1-knockout cells.

**Conclusion:**

The results suggest that legumain secretion from DJ-1-knockout cells and in mice increases through its increased expression and accumulation in membrane-associated vesicles.

## 1 INTRODUCTION

Asparaginyl endopeptidase, also known as legumain (EC 3.4.22.34) belongs to the cysteine peptidase C13 family [1]. Legumain activity has been detected in various mammalian tissues, and strong legumain activity was observed in the kidney [2]. Legumain is highly expressed and degrades fibronectin when remodeling the extracellular matrix in proximal tubules of the kidney [3, 4], and it plays an important role in tumor growth/metastasis [5-9]. Functions of legumain in addition to those described above have been described in a recent review [10].

Recently, we found that transcription of the legumain gene is regulated by p53 [11] through DJ-1 [12]. DJ-1 is a novel oncogene [13] and a transcriptional regulator [14-21].

In this study, we found that legumain secretion into serum was increased in DJ-1- knockout mice, that legumain localization was altered in DJ-1-knockout cells compared to that in wild-type cells, and that legumain concentration in membrane-associated vesicles was increased in DJ-1-knockout cells. These results suggest that prolegumain secretion increases in DJ-1-knockout mice through its accumulation in membrane- associated vesicles.

## 2 MATERIALS AND METHODS

### 2.1 Materials

A mouse legumain ELISA kit was obtained from MyBioSource (San Diego, CA, USA). Anti-legumain antibody (goat) was purchased from Santa Cruz (cat. no. sc-47105, CA, USA). All other analytical-grade chemicals were purchased from Wako Pure Chemicals (Osaka, Japan).

### 2.2 Animals

Wild-type and DJ-1-knockout mice were described previously [18]. DJ-1-knockout mice and wild-type mice were housed under SPF conditions. Serum was isolated from wild-type and DJ-1-knockout mice at 23 weeks of age.

### 2.3 Cell Culture

Mouse embryonic fibroblast cells were cultured at 37ºC in a humidified atmosphere containing 5% CO_2_ using Dulbecco’s modified Eagle’s medium supplemented with 10% calf serum. The conditioned medium was collected from DJ-1-wild and -knockout cells.

### 2.4 Western Blotting and Antibodies

Proteins were separated on a 12.5% SDS-PAGE and subjected to Western blotting with anti-legumain (1:500), anti-DJ-1 (1:1000) and anti-fibronectin (1:1000) antibodies. The reaction mixtures containing these antibodies were incubated at 4°C for 12 hours. Proteins on the membrane were reacted with Alexa Fluor 680- (Molecular Probes, Eugene, OR, USA) or an IRDye 800-secondary antibody (Rockland, Philadelphia, PA, USA) and visualized by using an infrared imaging system (Odyssey, LI-COR, Lincoln, NE, USA).

### 2.5 Immunofluoresence

Mouse embryonic fibroblast cells were fixed with 4% paraformaldehyde for 15 min and then with 0.1% Triton X-100 for 10 min. The cells were reacted with an anti-legumain antibody (1:100) for 2 hrs. The cells were then reacted with an Alexa Flour 488-conjugated anti-rabbit IgG for 1hr. They were then observed under a fluorescent microscope (Biorevo BZ-9000, Keyence, Osaka, Japan).

### 2.6 Enzyme-linked Immunoassay

The serum levels of legumain were measured using a mouse legumain ELISA kit (MyBioSource, CA, USA) according to the manufacturer’s instruction manual.

### 2.7 Ethics Statement

All animal experiments were carried out in accordance with the National Institutes of Health Guide for the Care and Use of Laboratory Animals. The protocols were also approved by the Committee for Animal Research at Hokkaido University (permit number 08-0467).

### 2.8 Statistical Analyses

Statistical analyses were performed using analysis of variance (one-way ANOVA) followed by unpaired Student’s *t*-test. Data are expressed as means ± S.E.

## 3 RESULTS

### 3.1 Secretion of Legumain in DJ-1-knockout Cells and Mice

**Fig. (1):**
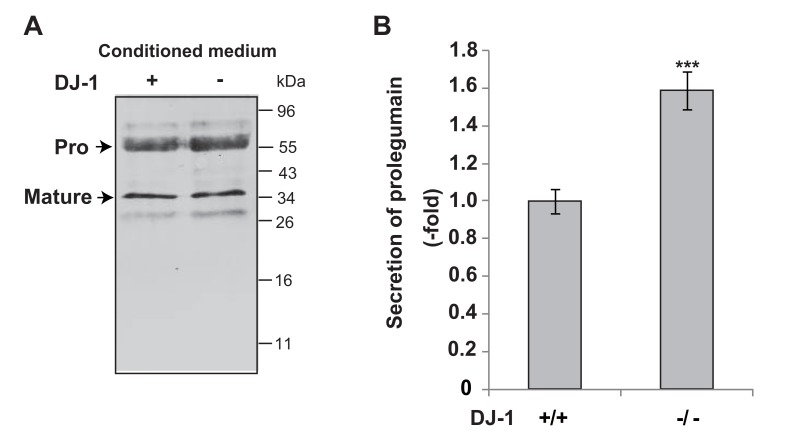
Secretion of prolegumain from DJ-1-knockout cells. Secretion levels of legumain in cultured media of DJ-1-knockout cells were examined by Western blotting (**A**). The relative secretion level of prolegumain is shown (**B**). Statistical analyses were carried out using Student’s t-test. Significance: **p<0.01, ***p<0.001.

To examine the secretion of legumain from DJ-1-knockout cells, cultured media were collected and subjected to Western blotting with an anti-legumain antibody. As shown in Fig. (**1A**), pro- and mature forms of legumain were detected in cultured media. The level of secreted prolegumain, but not that of matured legumain, from DJ-1-knockout cells was increased by about 1.6 fold compared to that from wild-type cells (Fig. **1B**).

### 3.2 Localization of Legumain in Wild-Type and DJ-1 Knockout Cells

An immunostaining experiment was carried out to examine the localization of legumain in wild-type and DJ-1-knockout cells. As shown in Fig. (**2**), the membrane loclization levels of legumain with dots were increased in DJ-1-knockout cells but not in wild-type cells.

### 3.3 Legumain Secretion into Serum From DJ-1-Knockout Mice

**Fig. (3):**
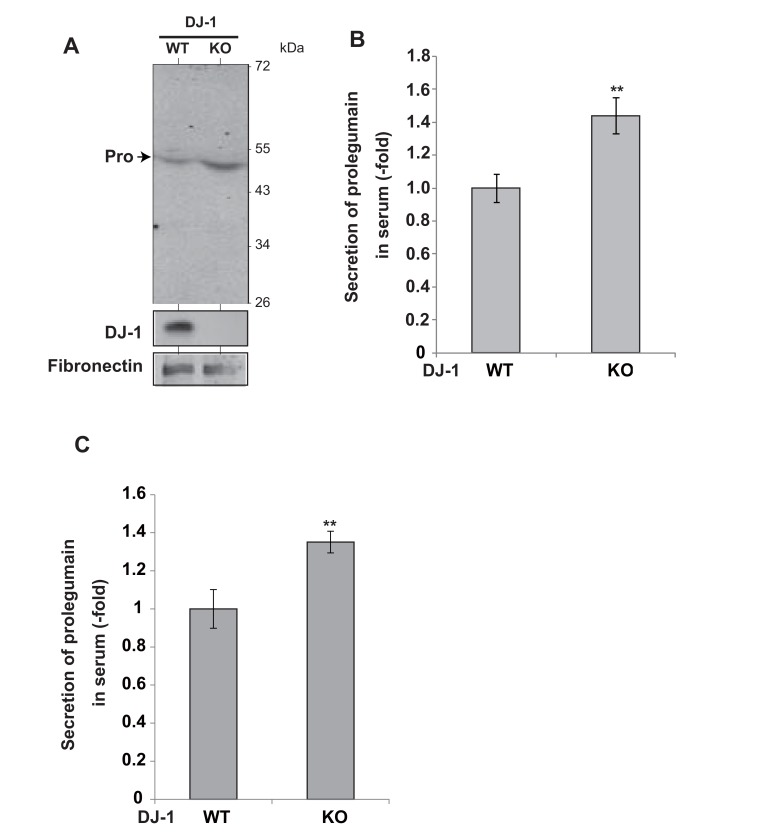
Secretion of prolegumain in serum from DJ-1-knockout mice. Secretion levels of legumain in serum from DJ-1-knockout mice were examined by Western blotting (**A**). The relative secretion level of prolegumain is shown (**B**). The relative secretion level of prolegumain was also examined by ELISA (**C**). Statistical analyses were carried out using Student’s t-test. Significance: **p<0.01, ***p<0.001.

To examine the secretion of legumain from DJ-1-knockout mice, serum was collected and subjected to Western blotting with an anti-legumain antibody. Prolegumain, but not the mature form of legumain, was detected in the serum (Fig. **3A**), and the legumain level in serum from DJ-1-knockout mice was increased by 1.5 fold compared to that in serum from wild-type mice (Fig. **3B**). Furthermore, ELISA analysis of the legumain level in serum showed a 1.4-fold increase in serum from DJ-1-knockout mice compared to the level in serum from wild-type mice (Fig. **3C**).

## 4 DISCUSSION

In this study, we showed that the secretion of legumain from DJ-1-knockout cells into the cultured medium and dot structures of legumain in DJ-1-knockout cells were increased. The secretion of legumain from DJ-1-knockout mice into serum was also increased. While both pro- and mature forms of legumain were observed in the conditioned medium of wild-type and DJ-1-knockout cells, only prolegumain was detected in serum from wild-type and DJ-1-knockout mice. These results indicate that legumain secretion is increased in DJ-1-knockout mice through its accumulation in vesicles and that secretory mature legumain is degraded immediately by a protease(s) in the blood. The secretion of prolegumain, but not that of mature legumain, was increased in the cultured medium of DJ-1-knockout cells and serum from mice. The molecular linker between DJ-1 protein and legumain secretion/maturation is not clear. Since DJ-1 is a cysteine protease belonging to the C57 family, the protein may induce legumain maturation by limited degradation. Furthermore, since the expression of legumain mRNA is regulated by DJ-1 through p53, a positive regulator of legumain transcription, DJ-1 protein regulates legumain secretion in an indirect manner. Since DJ-1 is a secretory protease, the secretion levels of other cysteine proteases in serum from DJ-1-knockout mice may be affected or increased to make up for the function of DJ-1 protease. Our previous studies showed that the expression of legumain was increased in DJ-1-knockout cells through transcriptional regulation by p53 [12] and that legumain expression was increased by doxolubicin-induced p53 in HCT116 cells [11]. Legumain expression is increased in tumor cells, and a high expression level of legumain accelerates invasion and metastasis of tumor cells [22]. Secretion of prolegumain also accelerates invasion and metastasis of tumor cells under acidic conditions [23]. Over-expression of cystatin E/M, a legumain inhibitor, suppresses melanoma cell invasion [24]. More cancer nodules were formed in the lungs of DJ-1-knockout mice than in wild-type mice at 3 weeks after B16F10 murine melanoma cells had been intravenously injected into mice [25]. Recently, we found that the level of legumain activity in the lungs of DJ-1-knockout mice was increased by about 75% compared to that in the lungs of wild-type mice [26]. Our present and previous findings suggest that secretion of prolegumain into the lungs of DJ-1-knockout mice is increased during metastasis of melanoma cells.

## CONCLUSION

In conclusion, prolegumain secretion in DJ-1-knockout mice and from cells is increased through its accumulation in membrane-associated vesicles.

## Figures and Tables

**Fig. (2):**
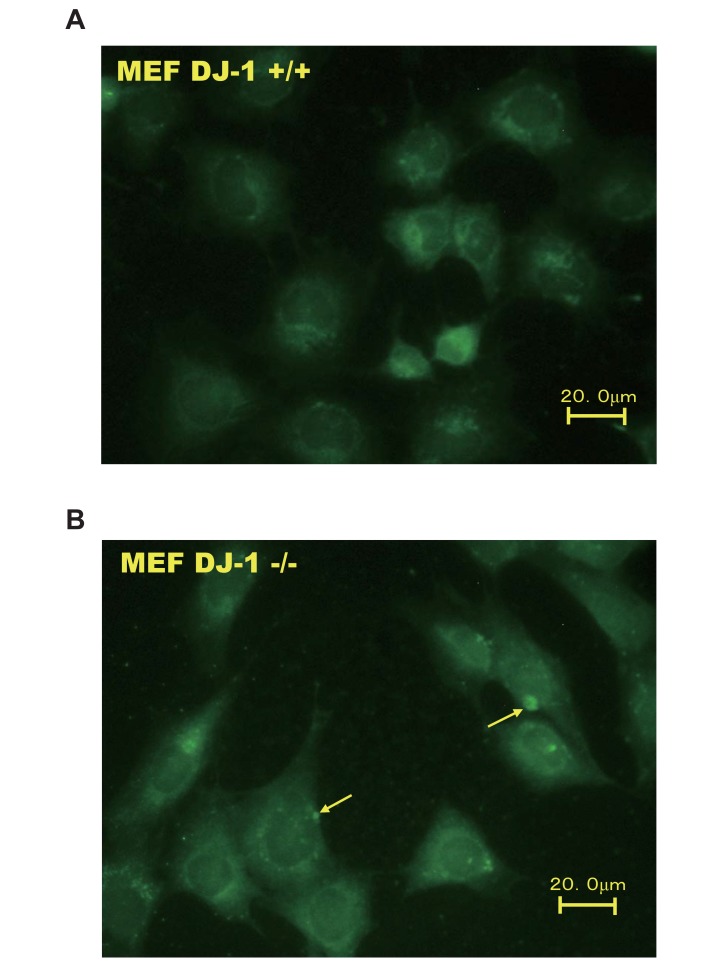
Changes of legumain localization in DJ-1-knockout cells. Wild-type and DJ-1-knockout cells were immunostained with an anti-legumain antibody and reacted with an Alexa Flour 488-conjugated anti-rabbit IgG for 1 hour. The magnification is 200x.

## LIST OF ABBREVIATION

ELISA: = enzyme-linked immunoassay
